# Surgical Steps of Gasless Transaxillary Endoscopic Thyroidectomy: From A to Z

**DOI:** 10.1155/2022/2037400

**Published:** 2022-12-10

**Authors:** Shujian Xu, Zhenlin Yang, Qingqun Guo, Weiwei Zou, Song Liu, Qiang Gao, Mengmeng Wu, Xingguo An, Yong Han

**Affiliations:** Department of Thyroid Surgery, Binzhou Medical University Hospital, No. 661 Huangheer Road, Bincheng District, Binzhou 256603, China

## Abstract

In the past 30 years, the incidence of differentiated thyroid cancer (DTC) has been increasing rapidly and has become one of the most common malignant tumors in females. Currently, the main surgical treatment for DTC is standard open thyroidectomy (SOT) via a traditional Kocher mid-cervical incision, but postoperative neck scarring was associated with significantly worse health-related quality of life (HRQOL) scores. To offer better cosmesis, robotic/endoscopic thyroidectomy via cervical, axillary, anterior chest, breast, postauricular, or transoral approaches have been developed over the past 20 years. In general, gasless transaxillary endoscopic thyroidectomy (GTET) has advantages in terms of convenience, clarity of vision, and aesthetic incision. The current work aims to provide a step-by-step description of GTET, supported by a high-quality, pictorial guide.

## 1. Introduction

In the past 30 years, the incidence of DTC has been increasing rapidly around the world and has become one of the most common malignant tumors in females [1–3]. Currently, the main surgical treatment is standard open thyroidectomy (SOT) via a traditional Kocher mid-cervical incision [4], but postoperative neck scarring was associated with significantly worse health-related quality of life (HRQOL) scores [5, 6], especially in African and Asian patients who have a tendency towards hyper-pigmentation and scar formation [7–9]. Minimally invasive video-assisted thyroidectomy (MIVAT) was first described in 1999 [10], and it has become a widespread technique for low- and intermediate-risk DTC [11–13]. Although the surgical incision of MIVAT is only about 2 cm, there are still a considerable number of patients with cervical scar hyperplasia [14–16]. A smaller incision in the neck was not associated with better patient satisfaction [17–19]. To offer better cosmesis, robotic/endoscopic thyroidectomy via cervical, axillary, anterior chest, breast, postauricular, or transoral approaches have been developed over the past 20 years [20–23]. In general, GTET has advantages in terms of convenience, clarity of vision, and aesthetic incision [24–26]. Previous reports have already described the surgical technique [27–29], but the details have not been provided and are still the prerogative of the expert surgeon. The current work aims to provide a step-by-step description of GTET, supported by a high-quality, pictorial guide.

## 2. Materials and Methods

This report has been reviewed and approved by the Ethics Committee of Binzhou Medical University Hospital, and informed consent has been obtained from the patient whose photos were used. All procedures performed in this study involving human participants were in accordance with the Declaration of Helsinki (revised in 2013).

**Figure 1:**
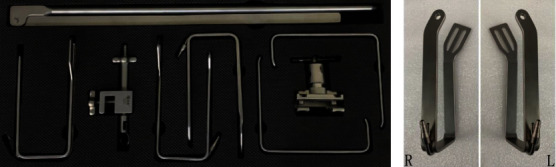
(a) The instrument for the GTET approach. (b) “U” type retractor with left and right side bend and suction pipe.

**Figure 2:**
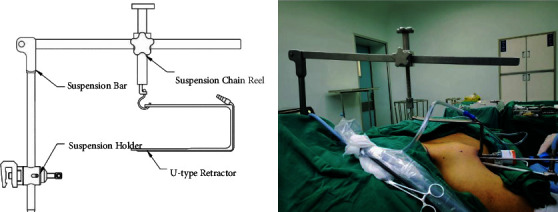
Schematic diagram (a) and intraoperative photos (b) of instrument.

The instrument consists of an inverted “L”-shaped bracket, a connector, and a “U”-shaped hook (Figures 1 and 2) (Hangzhou Kangji Medical Instrument Co., Ltd., China). The “U”-shaped hook has a suction tube, and the front end has a 30° bend (Figure 1(b)), which is fixed to the operation bedside (Figure 2).

## 3. Surgical Steps, Operating Techniques, and Hints and Pitfalls

### 3.1. Step 1: Body Position and Incision

**Figure 3:**
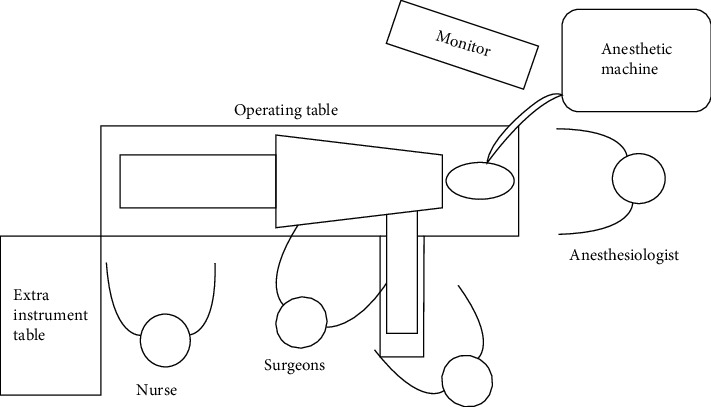
Operating room setup for the operation.

The layout of operators, assistants, anesthesiologists, and instrument nurses in the operating room is shown in Figure 3.

**Figure 4:**
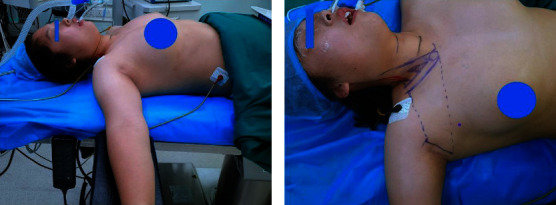
(a) Body position. (b) Subcutaneous tunnel marking.

The patient was in the supine position, with the neck and shoulders elevated, and the affected upper extremity abduction nearly 90° to expose the armpits (Figure 4(a)). The surgical incision is along the natural wrinkles of the axilla, and the length is about 5 cm. Mark the intersection of the horizontal line of the cricoid cartilage and the medial edge of the sternocleidomastoid muscle, and draw this point with the upper end of the axillary incision, that is, the upper boundary of the subcutaneous tunnel. Mark the ipsilateral sternoclavicular joint, and then connect the lower end of the axillary incision, which is the lower boundary of the subcutaneous tunnel. A 0.5 cm incision was made at the intersection of the anterior axillary line and the breast border for trocar placement (Figure 4(b)).

### 3.2. Step 2: The Establishment of Surgical Cavities

**Figure 5:**
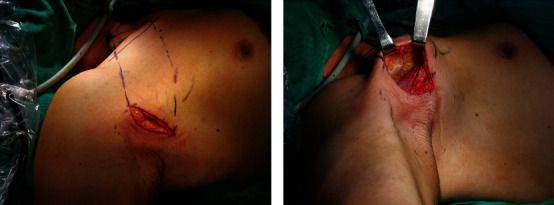
(a) The axillary incision was made. (b) The cavity is created under direct vision.

The dissociation range of the subcutaneous tunnel is from the axillary incision to the middle and lower 1/3 of the sternocleidomastoid muscle (SCM), and the dissociation level is located on the surface of the pectoralis major muscle fascia (Figure 5). When the subcutaneous tunnel near the clavicle is dissociated, a “U”-shaped” hook is placed to pull up the skin and subcutaneous tissue. A trocar is placed at the intersection of the anterior axillary line and the breast border (Figure 4(b)).

**Figure 6:**
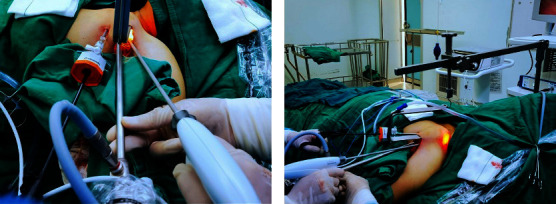
The endoscope and ultrasonic scalpel entered through the axillary incision, and the separation forceps inserted through the trocar.

The space was further dissociated using an ultrasonic scalpel under the observation of an endoscope (Figure 5). The ultrasonic scalpel and separating forceps were inserted through the axillary incision and trocar, respectively (Figures 6(a) and 6(b)).

**Figure 7:**
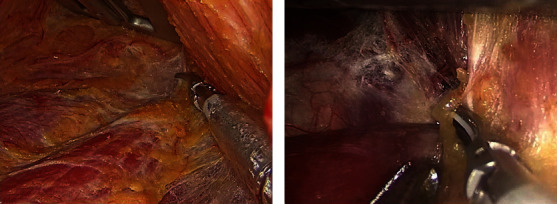
(a) The fascia and fat on the surface of the pectoralis major muscle were preserved. (b) The supraclavicular nerve was preserved.

Hints and Pitfalls: (I) Preserving the fascia and fat on the surface of the pectoralis major muscle (Figure 7(a)), which could reduce postoperative chest skin discomfort. (II) Beware of damage to the external jugular vein and its branches, which may cause troublesome bleeding. (III) Injury to the supraclavicular nerve should be avoided (Figure 7(b)); otherwise, postoperative numbness in the clavicle area would emerge.

**Figure 8:**
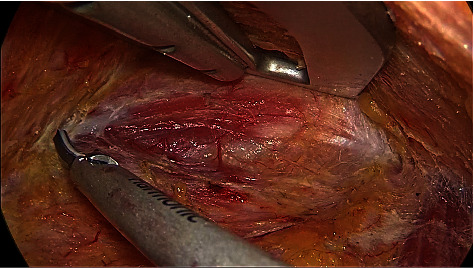
Fat and perforating vessels appeared in the gap between the sternal and clavicular heads of SCM.

The gap between the sternal and clavicular heads of the SCM was inserted with a “U”-shaped hook, and the cavity was further enlarged. The fascia between the internal jugular vein and the lateral margin of the sternothyroid was dissected, the potential gap between the sternothyroid and the true capsule of the thyroid was separated, and a hook was placed into the deep surface of the sternothyroid to lift the muscle. It is critical to explore the gap between the sternal and clavicular heads of SCM. It is characterized by the presence of fat and perforating vessels (Figure 8).

**Figure 9:**
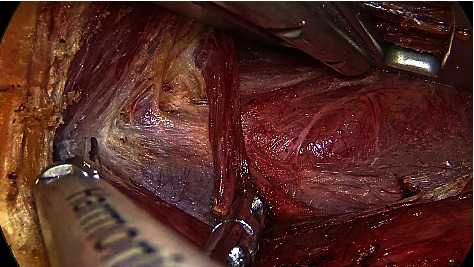
The space between the lateral border of the sternothyroid muscle and the internal jugular vein has been dissociated.

After the sternal head of the SCM was suspended, the gap was further expanded, and the omohyoid muscle was exposed. The spaces between the lateral border of the sternothyroid muscle and the internal jugular vein and the spaces between the deep surface of the sternothyroid muscle and the true capsule of the thyroid were opened sequentially (Figure 9). Be careful not to damage the internal jugular vein, which can lead to catastrophic bleeding. The key point is never to dissociate too much between the sternothyroid muscle and the thyroid gland. Once the space between the sternothyroid muscle and the thyroid is opened too much, it will cause the thyroid to fall, making it very difficult to remove the thyroid lobe and lymph nodes within the central compartment.

### 3.3. Step 3: Exposure, Dissection, and Protection of the Recurrent Laryngeal Nerve (RLN) and Central Neck Dissection

**Figure 10:**
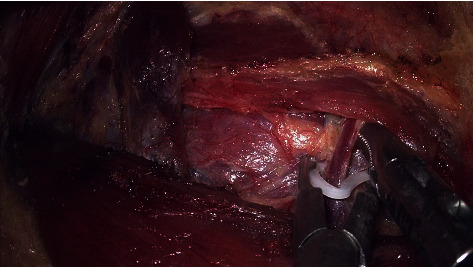
The middle thyroid vein was divided.

**Figure 11:**
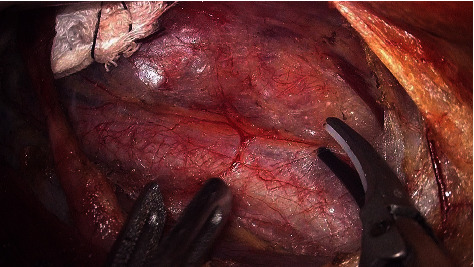
The medial edge of the carotid artery was dissected.

Once the strap muscles are retracted off the ventral surface of the thyroid, a preliminary dissection lateral to the thyroid is performed to identify the middle thyroid vein. The lateral thyroid region is opened by the division of the middle thyroid vein (MTV) (Figure 10). The medial edge of the carotid artery was dissected (Figure 11) to expose the external border of the central neck dissection (CND). The inferior parathyroid glands (iPTGs) locate in the area of CND and enjoy a more variable position in the adult neck, with most glands (approximately 80%) located in the area between the lower pole of the thyroid gland and the thymus (Wang, 1976). Thus, the iPTGs must be identified before CND. Proceeding dorsally, the next step is dissection of the plexus of inferior pole-related veins. As these veins are dissected close to the surface of the thyroid, the iPTGs are identified, swept away, and preserved, with dissection being medial to the upper cranial aspect of the iPTGs. The preservation of the parathyroid arterial supply is vital to preserving the viability of the parathyroid gland. It was referred to as the subcapsular procedure, and the point is based on the division of the thyroid arteries beyond the origin of the parathyroid arteries, close to the contact point with the thyroid parenchyma. Currently, iPTGs' preservation in situ during GTET remains the biggest challenge, and it is actually not easy to achieve.

**Figure 12:**
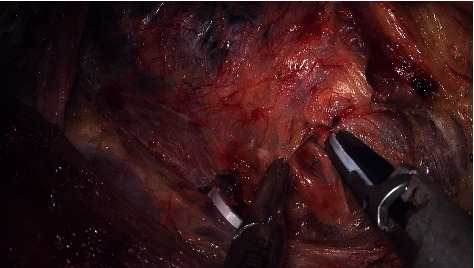
RLN was exposed in the tracheoesophageal groove.

A blunt separation in the middle and lower areas of the tracheoesophageal groove was performed until the RLN was exposed (Figure 12). The surface tissue is bluntly separated along the route of the RLN towards the nerve entry into the larynx. Meanwhile, the inferior thyroid artery is coagulated between the medial aspect of the RLN and the trachea.

**Figure 13:**
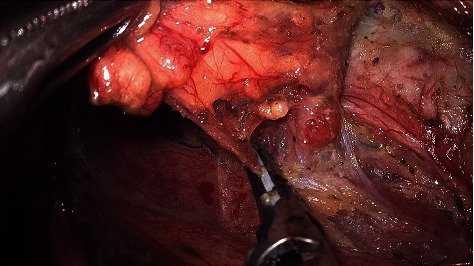
The paratracheal and pretracheal lymph nodes were dissected.

The central neck compartment is bounded superiorly by the cricoid cartilage, laterally by the carotid arteries, anteriorly by the superficial layer of the deep cervical fascia, posteriorly by the deep layer of the deep cervical fascia, and inferiorly by the suprasternal fossa. The midline is the outer edge of the healthy side of the trachea. Division of the sternothyroid muscle near the sternum can be helpful in gaining exposure of the suprasternal fossa and may improve the dissection of the lymph nodes at the thoracic inlet. Dissection was processed against the superficial layer of the deep cervical fascia; the deep or lateral lymph nodes of the RLN were dissected first, then lymph nodes superficial or medial to the RLN. Noting that the lymph nodes posterior to the RLN on the right need to be removed. Each branch of the RLN should be preserved as far as possible. The inferior thyroid veins were divided to expose the trachea, which was an important anatomical landmark of this operation. Finally, pretracheal lymph nodes were dissected along the surface of the trachea (Figure 13). The dissection proceeds laterally until the contralateral sternothyroid muscles and tracheal border are reached and finally detached from the thymus.

Hints and Pitfalls: (I) The more widespread use of energy devices (Harmonic Scalpel, LigaSure, etc.) and electrocautery (monopolar or bipolar) has likely led to an increased risk of thermal injury through improper application of heat to accomplish dissection around the RLN. The working head of the energy instruments should be ≥2 mm from the nerve, and the duration of the incision should be ≤3 s [30]. (II) As a novel injected suspension used during thyroidectomy in order to stain the thyroid gland and the lymph nodes while maintaining the anatomic color of the parathyroid glands and the laryngeal nerves, carbon nanoparticles may improve CND and parathyroid gland identification [31]. (III) It is very important to evaluate whether the iPTGs preserved in situ after CND are vascularized or not. We strongly recommend the fine-needle pricking test as a simple and reliable tool for evaluating the blood supply of the parathyroid gland [32]. Parathyroid auto-transplantation may represent the reasonable choice to restore parathyroid gland functionality in case of inadvertent removal or devascularization. (IV) It is particularly important to identify the brachiocephalic trunk division in preoperative computed tomography (CT). The absence of its visualization indicates Arteria Lusoria, followed by the nonrecurrent laryngeal nerve. An intraoperative nerve monitoring system can be used to assist in the identification and protection of the nonrecurrent laryngeal nerve, especially Type 2B [33]. (V) Sometimes it is difficult to distinguish between fat particles, lymph nodes, and parathyroid glands. If in doubt, a small piece of tissue should be removed for frozen section confirmation. Surgeons need to be alert to the possibility that metastatic lymph nodes could be mistaken for parathyroid glands and autoimplanted.

### 3.4. Step 4: Dissection of the Superior Pole and the External Branch of the Superior Laryngeal Nerve (EBSLN), Identification, and Protection of the Superior Parathyroid Gland

**Figure 14:**
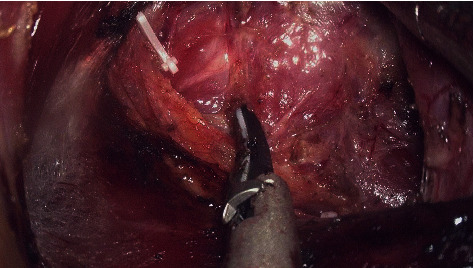
The EBSLN was encountered in the cricothyroid space.

The EBSLN should be exposed and identified before the coagulation of the superior thyroid vessels. The avascular space between the medial aspect of the superior thyroid pole and the cricothyroid muscle can be used as a surgical landmark where the nerve can be steadily encountered (Figure 14). Identification and dissection of this space are greatly assisted by traction of the thyroid in an inferior and lateral direction. The superior thyroid pole is pulled downwards to facilitate the skeletonization and coagulation of the superior thyroid vessels. The superior pole vessels should be sealed individually to optimize their control and avoid the risk of injury to the EBSLN. Posterior branches of the superior thyroid artery (STA) may contribute to the superior parathyroid glands (sPTGs) blood supply and should be reflected posteriorly and maintained if possible. Identification of the EBSLN can be difficult due to its variable course, and visualization alone was not enough because EBSLN display variation in their relationship with the STA. However, identification can be facilitated by using an intraoperative nerve monitoring system. The dissection was continued along the dorsal side of the upper pole of the thyroid. The sPTGs have relatively fixed locations, with 77% of them found at the cricothyroid junction posteriorly [34]. The sPTGs were dissected from the adjacent thyroid surface with a laterally-based vascular pedicle. The separation of the sPTGs and adjacent connective tissues without energy-based devices should be ensured. If the bleeding is minor, gauze compression can be very effective.

Hints and Pitfalls: (I) Skeletonization and coagulation of the vessels of the pedicle should take place against the thyroid capsule, which could effectively reduce the risk of injury to the EBSLN when it is adherent to or passing between the branches of the STA. (II) It is generally safe to use an ultrasound scalpel to coagulate the vessels of the pedicle. However, for large vessels, vascular clips or Hemolock may be used. (III) Selective division of the sternothyroid muscle can be helpful in gaining exposure of the superior pole and may improve visualization of the EBSLN.

### 3.5. Step 5: Processing the Ligament of Berry and en Bloc Resection of the Tumor

During a thyroidectomy, the highest risk of RLN injury exists around the ligament of Berry, where the RLN is closest to the thyroid. Generally, more than 2 mm of separation between the energy device and the RLN is needed to avoid thermal injury. One must be cautious that if the ligament of Berry has an element posterior to the RLN, then even judicious thyroid lobe retraction may be conveyed to the nerve and cause an upward bowing of the nerve up onto the lateral aspect of the trachea or even kinking of the nerve. Such retraction-induced positional changes to the nerve could lead to, at the very least, transient neuropraxia and are likely the cause of most cases of transient paralysis that surprise surgeons postoperatively. Data from neural monitoring studies show that RLN injury is most typically caused by stretch at the ligament of Berry, so great care and ongoing visual inspection here are extremely important and cannot be overemphasized [35, 36]. In all circumstances, as one retracts the thyroid lobe, one must keep the RLN in constant view to determine the effect of thyroid retraction on the nerve. This is especially true during ligament of Berry dissection.

The thyroid lobe and isthmus were dissected free from the trachea, and the pyramidal lobe and Delphian lymph nodes were dissected. So far, the thyroid lobe, isthmus, and central lymph nodes were removed en bloc, and the specimen was placed in a specimen bag and removed through a subcutaneous tunnel.

Hints and Pitfalls: (I) The ligament of Berry is dense, easily bleeds, and has a very close relationship to adjacent thyroid tissue. Careful transient bipolar cautery with fine-tipped forceps can be very useful. Cautery in this area without identification of the RLN may result in RLN injury. (II) In some patients, a small number of glands may be preserved at the ligament of Berry to ensure postoperative voice function and quality of life. (III) To avoid an inadvertent iatrogenic RLN thermal injury caused by energy-based devices, standard procedures for the safe use of these devices must be implemented.

**Figure 15:**
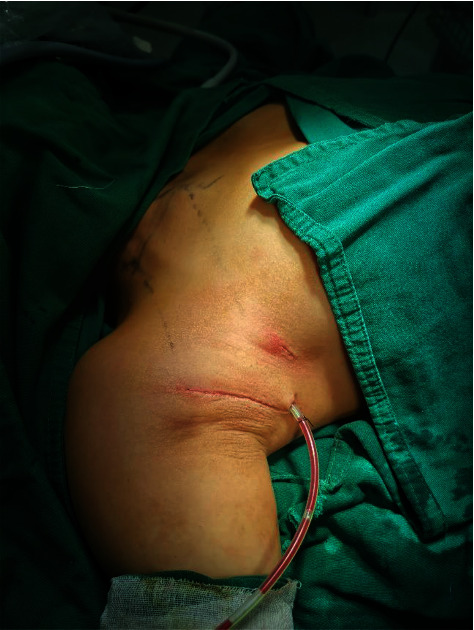
The drainage tube is left, and the incision is sutured.

The surgical cavity was irrigated thoroughly with distilled water. The SCM and supraclavicular fossa are prone to ooze blood; adequate hemostasis is also necessary. The drainage tube was placed through an axillary incision. Finally, the axillary incision was sutured with subcuticular absorbable sutures (Figure 15).

## 4. Discussion

There is a growing body of evidence suggesting that a scar in the anterior neck following thyroidectomy may have a significant quality-of-life effect on a patient with DTC [5, 37, 38]. Therefore, clinicians should pay attention to the psychological effects of scars on patients and take care to avoid a visible thyroidectomy scar. During the past two decades, there have been approximately 20 different thyroidectomy techniques proposed with the primary goal of avoiding a visible postoperative scar, such as moving the incision to a concealed area of the chest, axilla, or hairline. However, each approach has its own advantages, and it is difficult to completely avoid its inherent limitations.

In this article, we summarize the hints and pitfalls of GTET for DTC. The advantages of the procedure are as follows: (1) Injury to the RLN resulting in postoperative vocal disability is one of the most feared complications after thyroid and parathyroid surgery [39, 40]. A review of 27 articles and 25,000 patients showed that the average incidence of temporary postoperative vocal fold palsy was 9.8%, and the incidence of permanent RLN injury was 2.3% [41]. As in warfare, the key to success is “finding the enemy before the enemy finds us.” Currently, complete exposure of the RLN during thyroidectomy is accepted as the gold standard method for the preservation of the RLN [42, 43]. During thyroidectomy, the RLN can be identified using four different approaches, depending on the type of thyroid growth and the choice of the surgeon: there are lateral, inferior, superior, and medial approaches. The lateral approach is the most commonly used technique in primary thyroid surgery [42, 44]. The lateral and dorsal surfaces of the thyroid were reached first by GTET, which is conducive to the exposure and protection of RLN. The oral or transthoracic approach can be considered an endeavor that proceeds in a logical sequence of steps from the ventral (skin of the ventral neck) to the dorsal (toward the vertebral column) [45, 46]. Due to the obstruction of the thyroid lobe, it will be difficult to find RLN in the oral or transthoracic approach. However, it is easier to find RLN because both the thyroid and RLN are suspended in GTET. The incision is located under the natural folds in the armpit, where the skin is loose, scars are not easy to form, and the location is hidden, which can be covered by the axillary hair. The postoperative incision has a high cosmetic satisfaction rate and will not affect the patient's appearance or social communication. An open incision is used for GTET, and continuous negative pressure suction can realize the rapid exchange of air in the surgical cavity, which is conducive to the rapid discharge of high-temperature air and reduces the risk of thermal damage to the RLN. In addition, the removal of smoke is conducive to providing a clear vision and improving surgical safety and efficiency. The neck flap and strap muscle do not need to be separated, and the function of the anterior neck area is perfectly protected during GTET. There is no numbness and discomfort in the anterior neck, and there will be no tracheal-skin synkinesis during swallowing. The incision in the armpit is about 5 cm; therefore, the removal of the thyroid gland is easier, especially for larger tumors.

In order to avoid a visible scar, the GTET transfers the neck incision to the hidden armpit. The costs include having to rely heavily on energy-based devices for extensive tissue dissection, which poses greater challenges to operators. Besides, as with other remote access thyroid surgeries, GTET has failed to gain widespread acceptance in the surgical community because of the technical challenges, scepticism about oncological safety, and cost factors [47]. On the contrary, it is actually more invasive. Therefore, as with the other remote approaches, GTET is only applicable to patients who require no scar on the neck. For those who do not care for a visible postoperative scar, the preferred choice is the transcervical approach. In addition, it is more difficult to process contralateral glands and deal with central lymph nodes on the same side [48]. Therefore, it is more feasible for unilateral low- and intermediate-risk DTC.

## 5. Conclusion

Despite being a well-codified procedure, the literature on the detailed description of the GTET program is still scarce. This tutorial describes the GTET procedure step-by-step through high-quality pictures, specific tips, and tricks, aiming to promote more physicians to recognize and master the procedure.

## Data Availability

The data supporting the findings of this study are available on request.
